# Liquisolids as a platform for the formulation of cannabis tablets

**DOI:** 10.1016/j.ijpx.2026.100508

**Published:** 2026-02-24

**Authors:** Jan Appelhaus, Karl G. Wagner, Kristina E. Steffens

**Affiliations:** Department of Pharmaceutics, University of Bonn, Germany, Gerhard-Domagk-Str. 3, 53121 Bonn, Germany

**Keywords:** Cannabis, Liquisolid, Tableting, Stability, Liquisolid compact

## Abstract

Mesoporous silica-based liquisolids offer an effective method for transforming liquid or sticky actives, such as cannabis extracts, into free-flowing powders. This approach broadens the formulation strategies available for these actives, which are typically limited to liquid formulations or soft gelatin encapsulation, by facilitating the formulation of tablets. However, tableting liquisolids is challenging due to issues including low tensile strength, capping and delamination. Overcoming these challenges requires careful selection of excipients and tableting parameters to produce tablets with sufficient tensile strength while also maintaining high Overall Liquid Load. In our study, we utilized a 65/35 volumetric ratio of 0.75 mL/g liquid loaded Syloid® XDP 3050 silica and Vivapur® 101 as a binder/filler to formulate cannabis liquisolid tablets with a target THC dose of 10 mg per tablet. Findings indicated that a 4% concentration of Kollidon® CL-F was optimal for disintegration, while 0.5% magnesium stearate proved to be the most effective lubricant concentration. Utilizing a rotary press compaction cycle on a StylOne® compaction simulator, we found precompression around half the main compaction pressure allowed for maximizing tableting speed while maintaining high tensile strength and keeping tablet defects to a minimum. The resulting cannabis liquisolid tablets demonstrated a tensile strength of 1.58 N/mm^2^ and a high Overall Liquid Load of 38.4% (*V*/*V*). They exhibited acceptable disintegration time, friability, dissolution behavior and were proven to remain stable for 30 days at 40 °C and 0% humidity. Under refrigerated conditions, stability is predicted to be >3 years.

## Highlights

• A 65/35 ratio of Syloid® XDP 3050 to Vivapur® 101 enables tableting of viscous cannabis extracts.
• Precompression pressure and tableting speed are critical process parameters for liquisolid tablet production.
• Sticky and highly viscous extracts can be formulated into tablets using the proposed formulation and production strategy.
• Cannabis tablets are predicted to be stable for >3 years under refrigerated conditions.

## Graphical abstract

**Figure:**
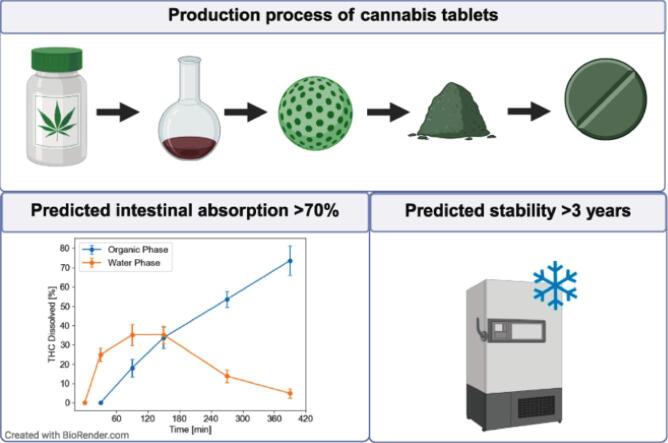

## 1 Introduction

In many countries, the medical use of cannabis has been legalized and liberalized, while some states have even decriminalized or legalized the use of cannabis for recreational purposes (Bahji and Stephenson, 2019). In practice today, cannabis medicines are mainly prescribed for chronic and cancer-related pain (McDonagh et al., 2022), multiple sclerosis (Nielsen et al., 2018) and anorexia/wasting (AbbVie Inc, 2017; Rosager et al., 2021). However, there are numerous other diseases for which cannabis medicines are used, or where beneficial effects are discussed in literature. These include but are not limited to cognitive impairment (Bilkei-Gorzo et al., 2017), migraine (Cuttler et al., 2020) or insomnia (Kuhathasan et al., 2022).

Over 400 active compounds in cannabis are described in literature (Pattnaik et al., 2022; Peschel and Politi, 2015), with the main actives being the psychoactive cannabinoid delta-9-tetrahydrocannbinol (THC) and non-psychoactive cannabidiol (CBD), which are produced upon heating the naturally more plentiful corresponding acids delta-9-tetrahydrocannbinolic acid (THCA) and cannabidiolic acid (CBDA) (ElSohly et al., 2017).

For treatment, physicians and patients can choose from a range of dosage forms. Dried cannabis flowers can be utilized in a manner similar to recreational consumption. However, this approach has the disadvantages of carrying social stigma (Reid, 2020) and making it challenging to determine and replicate a precise dose, as the smoking process is highly variable (Azorlosa et al., 1995; Lindgren et al., 1981). Consequently, cannabis extracts are frequently used. These extracts are produced from cannabis flowers using various extraction techniques, such as the use of different organic solvents. These can be used in vaporizers, which generally results in higher blood THC-levels compared to traditionally smoked cannabis (Spindle et al., 2018).

In contrast to inhalation, the onset of action is significantly delayed when cannabis medicines are administered orally, making this approach more suitable for long-term application. Cannabis extracts tend to be resinous/highly viscous and sticky in their undiluted form (Kebede et al., 2022), which limits processing and hinders the production of common oral dosage forms such as tablets. Currently, the only formulation option available for a single-dose oral dosage form is the manufacture of liquid-filled soft gelatine capsules like Marinol®, containing 2.5, 5 or 10 mg of synthetic THC (Dronabinol). Additionally, oral solutions like Syndros® and oromucosal sprays like Sativex® are also available.

As soft gelatin encapsulation is a highly specialized process, requiring dedicated machines and qualified employees, a cheaper and more mass producible single-dose oral dosage form for cannabis extracts and other resinous actives is desirable. A possible solution to this problem is the utilization of liquisolids. Here, a liquid or predissolved active is combined with adsorbent material to create an apparently dry, non-adherent powder, which can subsequently be formulated into a tablet or filled into a hard capsule.

This approach was first described by Spireas et al. (Spireas et al., 1992), who utilized microcrystalline cellulose in combination with colloidal silica as the adsorbent material. Since then, many authors report utilization of this technique to improve in vitro solubility of many APIs, including but not limited to carbamazepine (Javadzadeh et al., 2007), prednisolone (Spireas and Sadu, 1998), naproxen (Tiong and Elkordy, 2009) and simvastatin (Elkadi et al., 2017). Jaipakdee et al. already successfully formulated a cannabis extract into tablets using the liquisolid approach. Their work mainly focused on the dissolution performance of different liquid vehicles, with tablets produced using a hydraulic press. No lubricant or disintegrant was included in their formulation, leading to increasing disintegration times after storage. Furthermore, the compacts produced were notably large in size, due to the low maximum liquid load of the formulations used in the study (Jaipakdee et al., 2022).

Like the investigation by Jaipakdee et al.*,* studies of liquisolids mostly focus on formulation parameters like excipient selection, excipient ratios and the influence of various liquid vehicles. They mainly utilize very simple equipment like hydraulic presses, and even if advanced equipment like a rotary tablet press is used, important parameters such as tableting speed are rarely reported. Among >110 screened publications on “liquisolid compacts” found using scopus, no systematic evaluation of parameters like tableting speed, precompression pressure, dwell time, lubricant concentration and lubricant blending time could be identified, even as these parameters are critical to industrial scale production. To establish liquisolids as a viable industrial production technique, these factors must be investigated as an initial step toward assessing the scalability of liquisolid compacts.

In a previous study, we outlined the compression characteristics of liquisolids containing Syloid® XDP 3050 mesoporous silica and optimized binary mixtures of silica with common binders/fillers to achieve maximum Overall Liquid Load. A volumetric mixture of 30% Vivapur® 101 and 70% 0.75 mL/g liquid loaded Syloid® XDP 3050 was found to produce tablets of sufficient tensile strength while maintaining an Overall Liquid Load of 36–41% [V_Liquid_/V_Tablet_] with various liquid vehicles (Appelhaus et al., 2024). Continuously improving this optimized binary mixture, our study now aims to systematically evaluate the effects of disintegrant concentration, lubricant concentration, lubricant blending time, precompression pressure and tableting speed on liquisolid formulations. Subsequently, we intend to leverage these findings to formulate a sticky cannabis extract into a high drug load, mass producible and stable cannabis tablet containing 10 mg of THC.

## 2 Material and methods

### 2.1 Material

Dried cannabis flowers of the variety Pedanios 8/8 (Aurora Cannabis Enterprises Inc., Canada) were extracted using absolute ethanol (VWR International S.A.S., France). The extract was diluted using propylene carbonate (VWR International S.A.S., France). Syloid® XDP 3050 (Grace GmbH, Germany) was utilized as the mesoporous silica. The excipients microcrystalline cellulose (Vivapur® 101, JRS Pharma GmbH & Co. KG, Germany), crospovidone (Kollidon® CL-F, BASF SE, Germany) and magnesium stearate (Ligamed® MF-2 V, Peter Greven GmbH & Co. KG, Germany) were kindly donated by their respective manufacturers. HPLC grade acetonitrile (VWR International S.A.S., France) and trifluoroacetic acid (Merck KGaA, Germany) were used as eluents for the HPLC system. THC and CBD standard solutions (Sigma Aldrich Chemie GmbH, Germany) were analyzed to identify the respective substances. Sodium taurocholate hydrate (ThermoFischer GmbH, Germany), sodium chloride (Fischer Scientific, United Kingdom) and glacial acetic acid (VWR International S.A.S., France) were utilized to create the FeSSIF-V1 buffer solution. 1-Decanol (ThermoFischer GmbH, Germany) was employed as the acceptance layer in the BiPha+ apparatus. For the stability study, glass containers and respective twist-off lids (RIXIUS AG, Germany) as well as Microbag Tyvek® silica desiccant packages (Strobel GmbH, Germany) were utilized. Disodium mono‑hydrogen phosphate heptahydrate (Na2HPO4·7H2O, VWR International S.A.S., France) and monosodium dihydrogen phosphate monohydrate (NaH2PO4·H2O, VWR International S.A.S., France) were utilized to create phosphate buffer.

### 2.2 Formulation development

#### 2.2.1 General preparation of liquisolids

Liquisolids of a 0.75 mL/g Liquid Load Level were produced from propylene carbonate and cannabis extract mixtures thereof with Syloid® XDP 3050 as the absorbent material, utilizing the incipient wetness method. The neat propylene carbonate or propylene carbonate cannabis extract mixture was added to pre-dried silica and subsequently stirred with a spatula until no large lumps were visible. The resulting powder was mixed at 50 rpm in a Turbula® blender (Willi A Bachofen AG, Switzerland) to ensure full homogenization. This liquisolid was subsequently blended at the same rpm for 10 min with 30% Vivapur® 101 by volume to create a binary mixture. This volumetric ratio was found to be optimal for maximum liquid load in a previous study (Appelhaus et al., 2024).

If any additional excipients were added to this binary mixture, they were incorporated based on mass in relation to the volumetric mixture of liquisolids and Vivapur® 101, thereby maintaining a consistent silica-to-binder ratio. Any additional excipients, except the lubricant, were blended into the mixture simultaneously to Vivapur® 101.

#### 2.2.2 General compression process

The powder was compressed on a StylOne® classic (Medelpharm, France) compaction simulator equipped with 10 mm flat-faced punches using 450 MPa main compression pressure.

#### 2.2.3 Disintegrant concentration

Kollidion® CL-F was added to the optimized binary mixture created in Section 2.2.1 at concentrations of 0, 2, 4 and 6% [m/m] (Table 1) using a Turbula® mixer rotating at 50 rpm for 10 min. The blend was compressed according to Section 2.2.2 using the standard compaction cycle at 10% speed, manual die filling and lubrication. For each mixture, disintegration time of six tablets was measured according to Section 2.2.4. Likewise, the tensile strength of five tablets was recorded according to Section 2.2.5.

**Table 1:** Powder blends for disintegration time tested.

Excipient	Blend 1 [m/m]	Blend 2 [m/m]	Blend 3 [m/m]	Blend 4 [m/m]
0.75 mL/g Syloid® XDP 3050	76.9%	75.4%	73.8%	72.3%
Vivapur® 101	23.1%	22.6%	22.2%	21.7%
Kollidon® CL-F	0%	2%	4%	6%

#### 2.2.4 Disintegration time

Tablet disintegration times were measured using a DT50 disintegration time tester (SOTAX GmbH, Germany). The testing was conducted in accordance with Ph. Eur. chapter 2.9.1, Method A (Europäisches Arzneibuch 10.0–10.4, 2022), using deionized water heated to 37 °C. The measurements were performed for a maximum of 20 min to observe possible disintegration times close to but exceeding the 15 min limit specified in the Ph. Eur. for non-coated tablets.

#### 2.2.5 Tensile strength

After production, tablets were stored at 20 °C and 25–40% humidity for 24 h. A Mitutoyo Absolute ID C125B (Mitutoyo Corp., Japan) dial gauge was used to determine the height of the tablets. An AG 204 analytical balance (Mettler Toledo GmbH, Germany) was used to determine the tablet weight and a TBH210 (ERWEKA GmbH, Germany) was used to measure crushing force. The tensile strength was calculated according to *Fell and Newton* (Fell and Newton, 1970) (Eq. (1)).

(1) $$\mathrm{TS}=\frac{2*F}{\pi *d*h}$$

where TS is the tensile strength, F is the crushing force, d is the tablet diameter and h is the tablet height.

#### 2.2.6 Internal lubrication

To determine the effect of internal lubrication, 0, 0.5, 1 and 1.5% [*m/m*] of pre-sieved magnesium stearate was added to Blend 3 (Table 1) created in Section 2.2.3 (Table 2). A version of each Blend was mixed for 0.5, 1, 1.5, 2 and 2.5 min in a Turbula® mixer at 50 rpm. The sandwich method was used to ensure homogeneous mixing. Tablets were produced using the same tooling and main compression pressure described in Section 2.2.2, using manual die filling and the standard compression cycle at 10% speed. Ejection force was recorded from the internal force sensors of the tableting press. Tensile strength of five tablets was determined according to Section 2.2.5 for each Blend (Table 2) and mixing time combination.

**Table 2:** Powder blends derived from blend 3 for testing of internal lubrication.

Excipient	Blend 3 [m/m]	Blend 3B [m/m]	Blend 3C [m/m]	Blend 3D [m/m]
0.75 mL/g Syloid® XDP 3050	73.8%	73.4%	73.1%	72.7%
Vivapur® 101	22.2%	22.1%	21.9%	21.8%
Kollidon® CL-F	4%	4%	4%	4%
Ligamed® MF-2 V	0%	0.5%	1%	1.5%

#### 2.2.7 Precompression and tableting speed

To investigate the effects of tableting speed and precompression pressure, two mixtures, one containing 30% (*v/v,* Blend 3B*,* Table 3) and the other one containing a higher binder/silica ratio of 35% (*v/v,* Blend 3B2, Table 3) were produced according to the loading and blending technique described in Section 2.2.1, with the lubricant added separately using the sandwich method and 0.5 min blending time. Tablets according to described in Section 2.2.2. The tableting cycle simulated a Fette® 102i rotary press equipped with Euro B punches, operating at turret speeds of 20, 30, 40, and 50 rpm. The precompression was varied from 0 to 450 MPa in increments of 50 MPa. The die was filled using the standard StylOne® classic feeding shoe equipped with a square blade operating at 20% speed clock-wise. Five tablets were produced for each combination of turret speed and precompression level. After being stored for 24 h at 20 °C and 25–40% humidity, tablets were analyzed for tensile strength according to Section 2.2.5.

**Table 3:** Powder blend derived from blend 3B for testing of tableting speed and precompression.

Excipient	Blend 3B [m/m]	Blend 3B2 [m/m]
0.75 mL/g Syloid® XDP 3050	73.4%	69.3%
Vivapur® 101	22.1%	26.2%
Kollidon® CL-F	4.0%	4.0%
Ligamed® MF-2 V	0.5%	0.5%

### 2.3 Cannabis extraction and tableting

#### 2.3.1 Cannabis decarboxylation and extraction

Dry cannabis blossoms were spread thinly on a metal tray and placed into an UM-400 compartment dryer (Memmert GmbH & Co. KG, Germany) preheated to 120 °C. After 30 min the decarboxylated cannabis was removed from the dryer and approx. 10 g was added to a 100 mL round-bottom flask. For larger batches, the size of the flask was scaled proportionally to the amount of cannabis used. After cooling to room temperature, 10 mL of absolute ethanol was added for each gram of cannabis inside the flask. After 30 min in a Sonorex digitec ultrasonic bath (BANDELIN electronic GmbH & Co. KG, Germany), the residual plant material was filtered off and the ethanol was removed under vacuum at 40 °C in a Heidolph 2 rotary evaporator (Heidolph Scientific Products GmbH, Germany). To ensure the complete removal of residual ethanol, the extract was subsequently dried in a vacuum oven (Binder GmbH, Germany) at 40 °C for 12 h.

#### 2.3.2 Cannabinoid profile and HPLC quantification

The cannabinoid profile of the extract was measured using the HPLC method developed by Peschel and Politi (Peschel and Politi, 2015)*.* An Agilent Series 1100 HPLC System (Agilent Technologies Deutschland GmbH, Germany) equipped with a Nucleosil C18 column (5 μm, 4.6 × 250 mm, Macherey Nagel GmbH & Co. KG, Germany) as well as a Nucleosil C18 pre-coloumn (Macherey Nagel GmbH & Co. KG, Germany) was used for the measurements. Retention times of THC and CBD were identified using HPLC reference standards.

#### 2.3.3 Liquisolid preparation and tableting

Using propylene carbonate, the dried cannabis extract was diluted, resulting in a THC concentration of 100 μg/mL. The extract was then adsorbed onto pre-dried Syloid® XDP 3050 by adding the silica to the cannabinoid solution. The mixture was stirred with a spatula until no large lumps were visible. To ensure a homogeneous distribution, the powder was left in a Turbula® mixer at 50 rpm overnight. Additional excipients according to Blend 3B2 in Table 3, except the lubricant, were added and subsequently mixed for 10 min in a Turbula® blender at 50 rpm. After that, the lubricant was added using the sandwich method, and the mixture was blended for an additional 30 s. Finally, the mixture was compressed with a pre-compression pressure of 250 MPa and a main compression pressure of 450 MPa at a turret speed of 30 rpm, using a fill depth of 11.5 mm and 10 mm flat-faced punches. The die was filled by the StylOne® standard force feeder equipped with square blades operating clock-wise at 20% speed. Tablets produced were tested according to the procedures outlined in 2.2.4, 2.2.5 and 2.4.

#### 2.3.4 Antioxidant

THC, as part of the cannabis extract, is known to be sensitive to oxidation. Therefore, 1 mg/mL of 6-O-palmitoyl ascorbic acid was dissolved in propylene carbonate to serve as an antioxidant. This antioxidant-containing solvent was subsequently utilized to produce liquisolid tablets following the steps outlined in Section 2.3.3.

### 2.4 Cannabis tablet testing and stability

#### 2.4.1 Cannabinoid content

To determine the cannabinoid content, an accurately weighted amount of cannabis extract or a single tablet was placed into a 100 mL volumetric flask. About 80 mL of isopropyl alcohol was added and the flask was placed into an ultrasonic bath for 30 min. After cooling the liquid to room temperature, the flask was filled to 100.0 mL with isopropyl alcohol. An aliquot of the solution was filtered through a 0.2 μm PTFE syringe filter (Macherey Nagel GmbH & Co. KG, Germany) before HPLC analysis according to Section 2.3.2.

#### 2.4.2 Overall liquid load

The Overall Liquid Load (OLL) represents the percentage of liquid volume in relation to the overall tablet volume and thus represents a critical quality attribute for liquisolid tablets (Eqs. (2), (3), (4)).

(2) $$\omega_{S}=\frac{m_{l}}{m_{l}+m_{B}+\sum m_{n}}$$

(3) $$\omega_{l}=\frac{V_{l/g}*\rho_{l}}{\left(V_{l/g}*\rho_{l}\right)+1}$$

(4) $$\mathrm{OLL}=\frac{\left(\left(m_{\mathrm{Tab}}*\omega_{s}*\omega_{l}\right)÷\rho_{l}\right)}{V_{\mathrm{Tab}}}$$

where ω_s_ is the mass fraction of the loaded silica of the tablet, m_l_ is the mass of liquisolid in the formulation, m_b_ is the mass of binder used in the formulation and m_n_ is the mass of all additional components of the mixture. ω_l_ is the mass fraction of the liquid in the loaded silica, V_l/g_ the Liquid Load Level and ρ_l_ the density of the liquid.

#### 2.4.3 Residual ethanol content

The residual ethanol content was determined using a Focus® GC (Thermo Fisher Scientific, USA) connected to a TriPlus® auto-sampling unit (Thermo Fisher Scientific, USA) configured in headspace mode. The gas chromatograph was equipped with an FS-CS-624 capillary column (30 m × 0.32 mm, 1.8 μm, CS - Chromatographie Service GmbH, Germany). Samples were prepared by adding one precisely weighted tablet to 5 mL of phosphate buffer at pH 6.8, created by dissolving 13.124 g of disodium phosphate heptahydrate and 7.043 g of monosodium phosphate monohydrate in 1 L of distilled water. Subsequently, the samples were stored at room temperature for at least 2 h before measurement. Following incubation at 80 °C for 10 min, 1.0 mL of the gas phase was injected. The injector temperature was maintained at 220 °C with a split ratio of 1:25. Nitrogen gas served as the carrier at a flow rate of 2 mL/min. Throughout the measurement, the column oven temperature gradually increased from 60 °C to 80 °C at a rate of 2 °C/min. Detection was performed using a flame ionization detector set at 240 °C.

#### 2.4.4 Friability

Tablet friability was measured using a TA3R friability tester (ERWEKA Apperatebau GmbH, Germany). The testing was conducted in accordance with Ph. Eur. Chapter 2.9.7 (Europäisches Arzneibuch 10.0–10.4, 2022).

#### 2.4.5 Biphasic dissolution

Biphasic dissolution was performed using the BiPha+ apparatus designed by Denninger et al. This device contains four cylindrical vessels, three of which are designated for samples and one as a blank control. The vessels are maintained at a temperature of 37 °C using a water bath (Denninger et al., 2020). 50.0 mL of FeSSIF-V1 solution (Teleki et al., 2020) (Table 4) adjusted to a pH 5.0 and a precisely weighted tablet were added to each sample vessel to start the experiment. After 30 min, 50.0 mL of a decanol absorbance layer was introduced to simulate intestinal absorption. Samples of 1 mL were drawn from both phases of each vessel 30, 90, 150, 270, and 390 min after the start of the experiment. After each sample was taken, the volume drawn was replaced with fresh FeSSIF-V1 or decanol. Samples were analyzed for cannabinoid content according to Section 2.4.1.

**Table 4:** Composition of FeSSIF-V1 dissolution medium.

Component	Concentration [mM]
Taurocholate	15
Phospholipids	3.75
Sodium	319
Chloride	203
Acidic acid	144

#### 2.4.6 Stability

The stability of the formulations produced in Section 2.3.3 was tested under ICH Q1A (R2) accelerated conditions of 40 ± 2 °C and 75 ± 5% humidity in the dark for 90 days (International Conference On Harmonisation Of Technical Requirements For Registration Of Pharmaceuticals For Human Use, 2003). Tablets were stored both in an open container as well as in a closed container containing a package of silica-based desiccant. Stability for both open and closed storage was evaluated in terms of cannabinoid content (*n* = 5, Section 2.4.1), tensile strength (n = 5, Section 2.2.4), disintegration time (*n* = 3, Section 2.2.5) and biphasic dissolution (n = 3, Section 2.4.5). Samples were drawn after 0, 7, 30 and 90 days of storage.

Tablets containing 6-O-palmitoly ascorbic acid as an antioxidant were only evaluated in terms of cannabinoid content (n = 5, Section 2.4.1) at the same storage conditions and sampling intervals.

#### 2.4.7 Arrhenius equation model

To predict shelf life, the proposed liquisolid cannabis tablet formulation was stored at various temperatures; the tablets produced according to Section 2.3.3 were subjected to stability testing at 50 ± 2, 60 ± 2, and 70 ± 2 °C in closed containers containing a silica-based desiccant. The cannabinoid content (n = 5, Section 2.4.1) was measured after 7, 14, 21, and 28 days of storage. The time until a “significant change” in drug content, defined as a reduction greater than 5%, was calculated using the Arrhenius equation (Eq. (5)), where k is the reaction rate constant, T is the absolute temperature, A is the frequency factor, EA is the molar activation energy, and R is the universal gas constant.

(5) $$k=A*e^{-\frac{E_{A}}{R*T}}$$

## 3 Results

### 3.1 Formulation prescreening

**Fig. 1:**
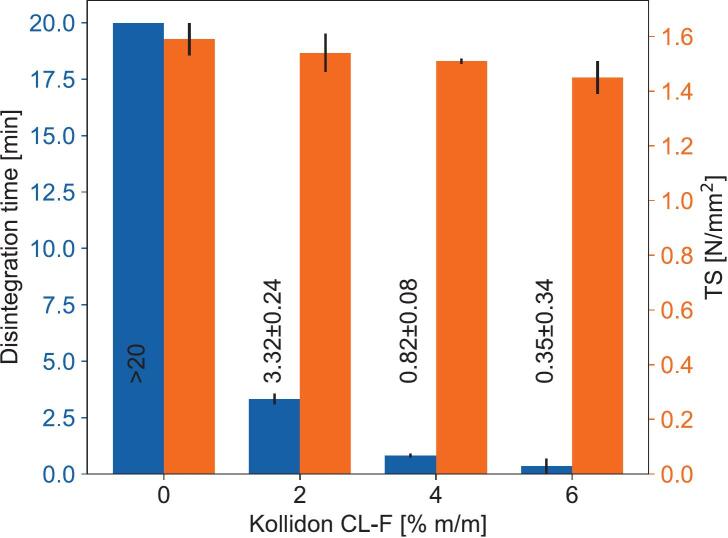
Disintegration time and tensile strength (TS) dependent on disintegrant concentration.

As shown in Fig. 1, the disintegration times of liquisolid tablets progressively become shorter with increasing concentrations of Kollidon® CL-F. The tensile strength only slightly decreased as the concentration of Kollidon® CL-F increased. All tablets, except for those containing 0% disintegrant, met the 15 min disintegration time limit set by the Ph. Eur. for non-coated tablets. A disintegrant concentration of 4% (m/m) was thus utilized in all further experiments to ensure fast and complete disintegration.

**Fig. 2:**
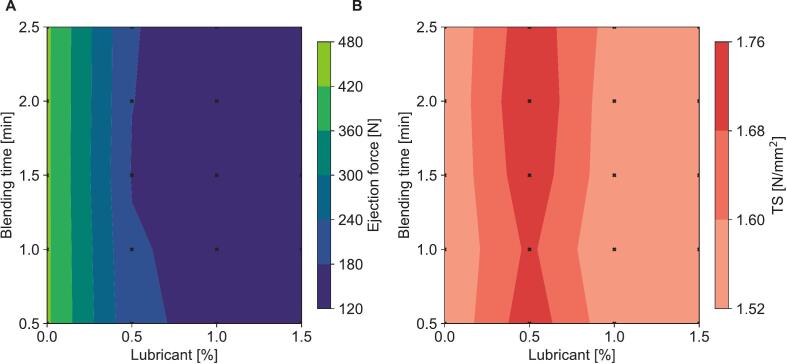
Contour plots of ejection force (A) and tensile strength (TS, B) against lubricant concentration and blending time in a Turbula® mixer after compression on a StylOne® classic using the standard compression cycle at 10% speed and a main compression of 450 MPa.

Increased lubricant concentration decreased the ejection force of the produced tablets, as can be seen in Fig. 2A. Tensile strength showed a maximum at a lubricant concentration of 0.5% (m/m), as can be seen in Fig. 2B. The blending time of the lubricant, however, had no effect on the ejection forces or tensile strength within the ranges tested in this study. A lubricant concentration of 0.5% (m/m) and blending time of 0.5 min was chosen for further experiments to maximize tensile strength.

**Fig. 3:**
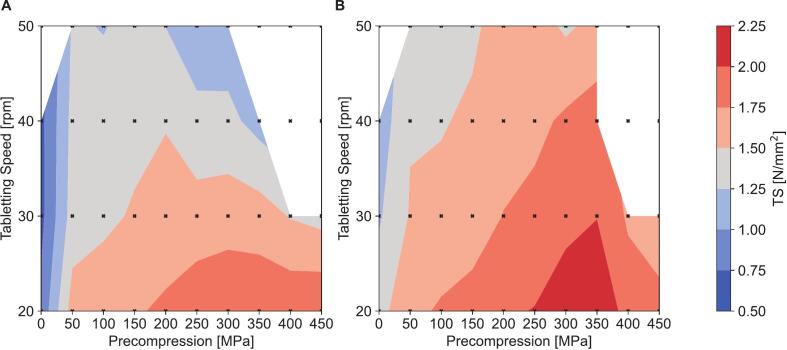
Tensile strength (TS) of blend 3B (A, Table 3) and blend 3B2 (B, Table 3) depending on tableting speed and precompression pressure on a StylOne® classic utilizing the Fette® 102i compression cycle and a main compression pressure of 450 MPa. White areas indicate tablet defects like capping or delamination for >20% of tablets produced.

The influence of simulated turret speed and precompression pressure on tensile strength and tablet defects was found to be considerable. As shown in Fig. 3, the tensile strength of the tablets generally increased with higher precompression pressure, reaching a maximum around 250 MPa. A pronounced increase was observed when transitioning from 0 MPa precompression pressure to 50 MPa. Conversely, increased turret speeds generally reduced tensile strength and increased the risk of tablet defects, mainly in the form of capping and/or delamination. These effects were very pronounced at high turret speeds in combination with either very low or very high precompression pressures. Increasing the binder content in the tableting mixture by approximately 5% (*v*/v; Table 3), increased tensile strength across the entire range of tested precompression pressures and turret speeds by about 0.25 N/mm^2^. Meanwhile, the increase in binder content only had minor impact on tablet defects, which persisted at high turret speeds in combination with very high or low precompression pressures.

### 3.2 Cannabis extraction, tableting and stability

The extraction of 80 g of cannabis flower yielded 14.2 g of raw cannabis extracts, giving a drug extract ratio of about 1:5. HPLC measurements revealed the THC concentration to be 22.8%, while the CBD concentration was measured at 28.0%. Tablets produced with antioxidant contained 10.0 ± 0.26 mg of THC on average, while tablets without antioxidant contained 9.58 ± 0.14 mg. Tablet characteristics measured as per Section 2.4 on the day of production can be found in Table 5.

**Table 5:** Characteristics of tablets without antioxidant directly after production.

Tablet characteristic	Value ± standard deviation
THC content	9.58 ± 0.14 mg
CBD content	12.37 ± 0.25 mg
Tensile strength	1.58 ± 0.13 N/mm^2^
Overall Liquid Load	38.4 ± 0.6%
Disintegration time	2.07 ± 0.41 min
Residual ethanol content	480 ± 6 ppm
Friability	0.49%
THC partitioned during dissolution	72.1 ± 8.5%

**Fig. 4:**
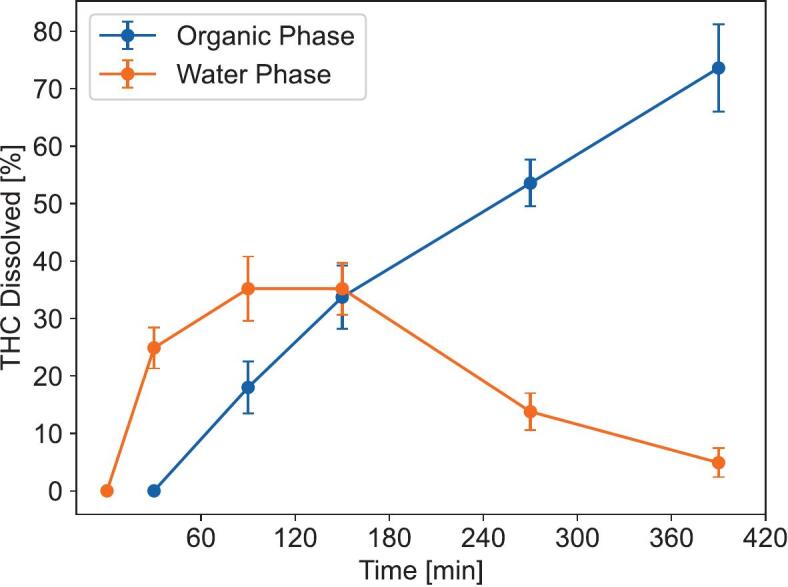
Biphasic tetrahydrocannabinol (THC) dissolution profile of cannabis tablets 24 h after production (*n* = 6) using FessiF at pH 5.0 as the aqueous and decanol as the organic phase.

For an estimate of potential intestinal absorption under fed conditions of the cannabis tablets a biphasic dissolution was performed (Fig. 4). In the BiPha+ apparatus, THC and CBD first dissolved into the biorelevant FeSSIF-V1 medium. Subsequent to the addition of the decanol absorption layer after 30 min, the dissolved cannabinoids partitioned into the decanol absorption layer due to their lipophilicity. By end of the test (390 min) 72.1% of THC partitioned to the decanol absorption phase.

**Fig. 5:**
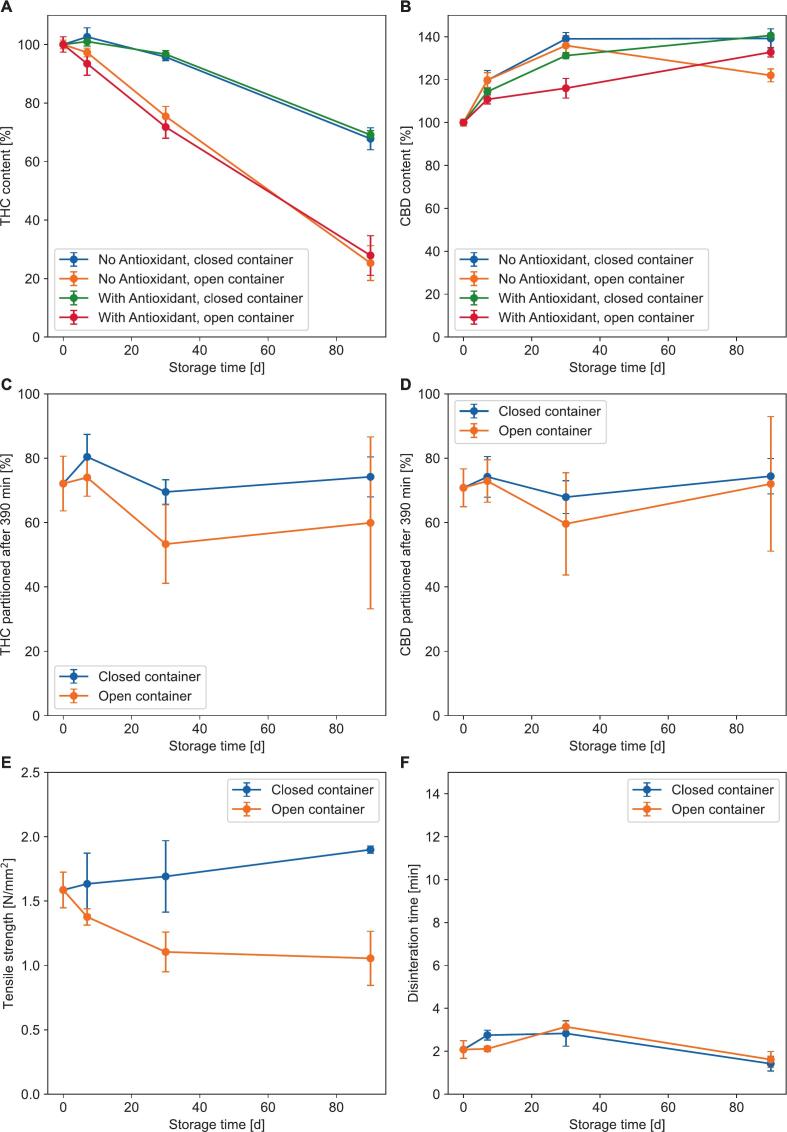
Stability of cannabis tablets under storage at 40 °C and 75% humidity with respect to THC and CBD content (A and B, *n* = 3), total amount of cannabinoid partitioned into the organic phase (C and D, n = 3), tensile strength (E, *n* = 5) and disintegration time (F, n = 3).

The stability of cannabis tablets was evaluated under ICH Q1A (R2) accelerated conditions in terms of cannabinoid content, dissolution, tensile strength, and disintegration time. In open storage conditions, the THC content experienced a significant decline after about 7 days. In contrast, THC levels in closed containers remained stable for the first 30 days, according to the ICH Q1A (R2) stability definition (Fig. 5A). The CBD content of the tablets increased to approximately 130%, regardless of the packaging (Fig. 5B). Regarding tensile strength, tablets stored in open conditions showed a decrease, while those kept in closed storage exhibited a slight increase (Fig. 5E). Disintegration times for the tablets in both storage scenarios remained well within the limits outlined by the European Pharmacopoeia (Fig. 5F). Tablets under closed storage conditions partitioned about 70% of the total contained THC into the absorption layer during dissolution tests (Figs. 5C, S1, S2) over the entire storage period, while tablets in open storage showed higher variance in dissolution behavior (Fig. 5C). The total amount of CBD partitioned consistently remained around 70%, irrespective of the packaging used (Figs. 5D, S3, S4).

**Fig. 6:**
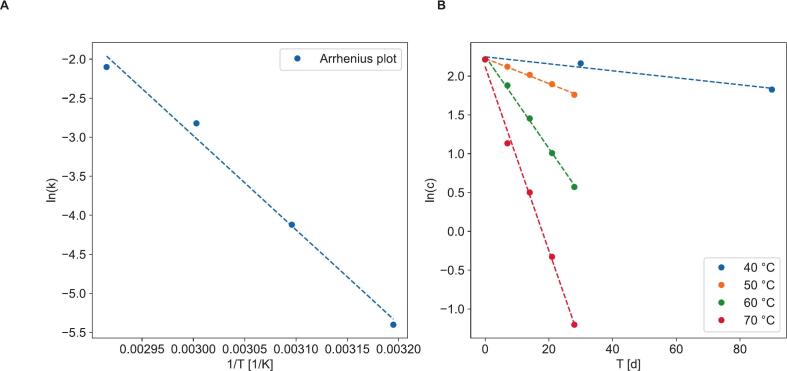
Arrhenius plot (A) and determination of the reaction kinetics (B) for THC degradation in liquisolid cannabis tablets at elevated temperature.

Experimental data collected during at 50, 60 and 70 °C and stability testing at 40 °C demonstrated that the thermal degradation behavior of THC is best described by first-order reaction kinetics (Fig. 6B, R^2^ > 0.99 for 50–70 °C). The Arrhenius equation was used to estimate the time until significant change in THC drug content in tablets stored at 25 °C and 5 °C, representing the long-term storage temperatures specified in ICH Q1A(R2) for general and refrigerated storage, respectively (Fig. 6A, Table 6) (International Conference On Harmonisation Of Technical Requirements For Registration Of Pharmaceuticals For Human Use, 2003).

**Table 6:** Projected time until a significant change in THC concentration.

Temperature	Projected THC stability
25 °C	73 days
5 °C	3.7 years

## 4 Discussion

Shown by the study of Jaipakdee et al., disintegration time is critical when formulating liquisolid tablets, because of possibly increasing disintegration times during storage (Jaipakdee et al., 2022). Thus, even though tablets containing 2% disintegrant would comply with the 15 min threshold set in the Ph. Eur. we chose a 4% disintegrant concentration for further studies to act as a safety margin upon storage. The impact of the chosen disintegrant on the tensile strength was found to be minor, with a given Kollidon® CL-F concentration in percent (*m/m*) decreasing the tensile strength by about the same percentage amount (Fig. 1).

Previous research has identified microcrystalline cellulose, specifically Vivapur® 101, as the most effective binder/filler for the production of liquisolid tablets with Syloid® XDP 3050, demonstrating both high tensile strength and high Overall Liquid Load. However, the research relied on external magnesium stearate for lubrication (Appelhaus et al., 2024), which may cause challenges in pharmaceutical production due to regulatory issues. Since microcrystalline cellulose is known to significantly decrease in binding performance with increasing lubricant concentrations and extended mixing times (Kikuta and Kitamori, 1994), it seemed essential to select appropriate parameters for the addition of magnesium stearate. While the concentration of lubricant had a noticeable influence on ejection force, the blending time appeared to have no significant effect as long as blending times were kept below 2.5 min (Fig. 2B). The differences in tensile strength among various lubricant concentrations were minimal, with 0.5% lubricant yielding the best results. This was unexpected, as the addition of internal lubricants typically results in a stagnation or decrease in tensile strength rather than an increase (Jarosz and Parrott, 1984). One possible explanation for this phenomenon is that the absence of lubricant may introduce minor defects due to high ejection forces, leading to reduced tensile strength. Therefore, a lubricant concentration of 0.5% (m/m) and a mixing time of 0.5 min were selected for all subsequent formulations.

Precompression pressure and tableting speed are critical factors in pharmaceutical production. Although there is currently limited information on the behavior of liquisolids in simulated or actual rotary tablet presses, existing research indicates significant impact of tableting speed and precompression pressure on tensile strength (Glišić et al., 2025; Woyna-Orlewicz et al., 2025; Zakowiecki et al., 2023). Our study confirmed that tensile strength is dependent on both tablet compression speed and precompression pressure. Generally, tensile strength decreased at higher tableting speeds, while the optimal precompression pressure was found to be between 200 and 300 MPa at constant main compression pressure of 450 MPa. At high tableting speeds (>40 rpm, linear speed of >0.6 m/s), we observed capping and delamination at very low (<50 MPa) or very high (>350 MPa) precompression pressures (Fig. 3). Apparently, the liquid loaded silica particles, which are not contributing to the bond formation in the tablet, were in fact impeding the plastic flow of MCC used as a filler binder. We hypothesize that the high precompression and main compression pressure were partially compensating for that effect below a limit of 350 MPa. A further explanation for these issues is air entrapment, as higher tableting speeds can significantly increase the air pressures exerted during compression (Klinzing and Troup, 2019). In our study, the mesoporous silica was loaded with only 0.75 mL/g of liquid, leaving 0.75 mL/g of air trapped within the silica's pores, which needs to be expelled during the compaction process. Although incorporating more liquid to address this problem is possible, this considerably decreases the Overall Liquid Load achievable at a given tensile strength, as previous research has shown (Appelhaus et al., 2024). Therefore, if the aim is to maximize tableting speed for a product that requires only a small amount of liquid, increasing Liquid Load Level to achieve higher tableting speed without capping issues might be an option. However, since liquisolid tablets are typically already bulky due to low overall drug loads, we decided not to pursue this option. Prolonged dwell time using Euro D tooling, Euro B tooling with an optimized punch head geometry or a tablet press offering an air compensator should mitigate the speed dependency of tableting liquisolid tablets.

While blend 2B and blend 2B2 (Table 3) performed similarly overall, the higher binder to silica ratio in blend 2B2 enabled higher tableting speed while maintaining acceptable tensile strength. Depending on process requirements, the target tensile strength is generally chosen between 1.0 and 1.7 N/mm^2^ (Pitt and Heasley, 2013). For our study, we thus chose a tensile strength of 1.5 N/mm^2^ as a target value. Because of the higher tensile strength, we chose blend 2B2 with a precompression pressure of 250 MPa as the basis for the production of our cannabis tablets.

Cannabis tablets produced in our study were of sufficient mechanical strength and had a high Overall Liquid Load, with disintegration time and friability below the threshold set in the Ph. Eur. Residual ethanol content is well below the 5000 ppm threshold set in the ICH Q3C (R9) guidelines (International Council for Harmonisation of Technical Requirements for Pharmaceuticals for Human Use, 2024). However, the tensile strength achieved by the cannabis tablets differed from the strength achieved by pure propylene carbonate tablets (1.58 N/mm^2^ vs. 1.82 N/mm^2^) using the same tableting parameters during the formulation pre-screening (Table 5, Fig. 3B). The difference is statistically significant (Welch-Test, *p* = 0.05) but not critical to the overall formulation as the tensile strength of the cannabis tablets remained above the target value of 1.5 N/mm^2^. Changes in the liquid vehicle are known to have similar effects in terms of absolute tensile strength reduction/increase to the one seen in this study (Appelhaus et al., 2024), but the mechanism behind this phenomenon is currently unknown and warrants further research.

Because THC and CBD are poorly water soluble, any cannabis extract released from the tablets might phase separate from an aqueous dissolution medium, preventing accurate quantification of cannabinoid release from the tablet. Thus, selecting an appropriate dissolution system is a key factor for the evaluation of the tablets performance. Using USP Type II based systems with synthetic emulsifiers like polysorbates is an option, but these strong emulsifiers might influence the dissolution process. While using a biorelevant medium like FeSSIF containing natural emulsifiers like bile salts can alleviate this problem, the large 900 mL volume of the USP II apparatus is also not representative of the in vivo conditions. Thus, we chose to employ the BiPha+ apparatus developed by *Denninger* et al. with 50 mL of FeSSIF-V1 medium at pH 5.0 as the aqueous phase and 50 mL of decanol as an absorption layer, which already demonstrated its ability to accurately predict in vivo performance for several poorly water soluble natural products formulated using different approaches (Brenner et al., 2023, Brenner et al., 2024). The results predict intestinal absorption exceeding 70%, suggesting THC bioavailability of 8–16% from the extract tablets, which is comparable to that of marketed soft gelatin capsules including synthetic THC. Oral cannabinoid bioavailability for these is given at 10–20% with 90% intestinal absorption, primarily due to extensive hepatic first-pass metabolism (AbbVie Inc, 2017).

The THC content of the tablets was stable for 30 days at 40 °C and 75% r.h. according to the definition of ICH Q1A(R2), which defines “significant change” as drug content change >5% with respect to the initial assay. After 90 days of storage, however, the THC content had decreased significantly with only 70% of THC remaining. The addition of antioxidant did not seem to significantly influence the stability of THC in our study, even though oxidation is known to be a significant cause for THC degradation (Fig. 5A) (Fairbairn et al., 1976). A likely explanation for this is the low amount of 0.1 mg of antioxidant in contrast to the 10 mg of THC present per tablet, which probably provided insufficient protection from oxidation. Further studies with higher concentrations are required to conclusively determine the effect of antioxidant on this formulation. CBD content significantly increased according to the ICH definition after 7 days of storage (Fig. 5B), most likely due to insufficient decarboxylation of CBDA in the original cannabis material. This was predictable, since our decarboxylation process was optimized for THCA while the decarboxylation rate of CBDA is known to be slower than that of THCA (Seo et al., 2022). While increasing CBD upon storage is undesirable for a commercial product, we chose to accept this in order to avoid the THC degradation associated with continued heating of the cannabis required for complete CBD decarboxylation. Commercialization of this cannabis formulation would thus require further optimization of the cannabinoid decarboxylation process.

During our stability testing, tensile strength of tablets in open containers fell below the target value of 1.5 N/mm^2^ defined in our study, while the tensile strength of those stored in closed containers increased slightly from 1.6 N/mm^2^ to 1.9 N/mm^2^ (Fig. 5E). The addition of a strong disintegrant to the liquisolid tablet solved the problem of increasing disintegration times upon storage or at higher tensile strength observed by Jaipakdee et al.*,* as the disintegration time remained below four minutes across the entire storage period (Fig. 5F).

Application of the Arrhenius model enabled projection of THC content stability under both general and refrigerated storage conditions. General storage of cannabis tablets at 25 °C is not recommended, as the THC content is predicted to deviate significantly from specification after only 73 days. In contrast, refrigerated storage at 5 °C is projected to maintain specification for 3.7 years. This observed stability aligns with marketed drug products containing cannabis extracts or THC, which are typically stored under refrigeration. For example, a marketed oromucosal spray containing a cannabis extract (Sativex®) specifies a shelf-life of 2 years at 2–8 °C (Sativex Oromucosal Spray - Summary of Product Characteristics), while a generic THC capsule specifies a shelf-life of 24 months at higher temperatures of 8–15 °C (Dronabinol Capsules 10 mg - Product details).

Comparing the produced cannabis tablets to a well performing formulation reported by Jaipakdee et al. (CS–PEG–c80) revealed that the tablets produced according to our method carry 100 mg of liquid per 300 mg of tablet weight, while using the formulation reported by Jaipakdee et al. would result in a final tablet weight of 673 mg for the same amount of liquid. Our high liquid load can be explained by the use of a mesoporous carrier in combination with microcrystalline cellulose binder/filler instead of using cellulose as a carrier. The final tensile strength recorded for both formulations was roughly equivalent (Our formulation 1.59 N/mm^2^ vs. Jaipakdee et al. 1.46 N/mm^2^), even as equipment and production parameters differed substantially, with our setup more closely resembling industrial production. Compared to our formulation, the formulation reported by Jaipakdee et al. showed prolonged disintegration times (2.07 min vs. 30.9 min), exemplifying the importance of disintegrant in optimal liquisolid formulations.

## 5 Conclusion

Previous research conducted by our group demonstrated the significance of binder selection in formulating mesoporous silica based liquisolids. This study furthermore emphasized the critical roles of precompression pressure and tableting speed in the development and scale up of liquisolid tablets. Both are essential for overcoming the poor tableting behavior of mesoporous silica while maintaining sufficient tensile strength. To achieve optimal results, a precompression pressure of approximately half of the main compression pressure was found to be ideal. Slower tableting speeds generally improved tensile strength and reduced tablet defects, but also decreased production rate. The selection of an optimal precompression pressure allows for maximizing the production rate while ensuring sufficient tensile strength and a high Overall Liquid Load. Using these insights, we successfully formulated a sticky cannabis extract into cannabis tablets. The obtained tablets demonstrated good disintegration and dissolution behavior, while maintaining a tensile strength greater than 1.5 N/mm^2^, with a high Overall Liquid Load of 38.4%, i.e. 10 mg THC in a 3.7 mm high flat-faced tablet of 10 mm diameter weighing around 375 mg. In a subsequent stability study, we found that tensile strength, disintegration time, and dissolution behavior remained stable for 90 days at 40 °C when packaged air-tight with a desiccant. However, THC content was only stable for 30 days under the same conditions. Application of the Arrhenius model predicted THC content to be within specification for 3.7 years at refrigerated storage conditions.

## CRediT authorship contribution statement

**Jan Appelhaus:** Writing – original draft, Visualization, Software, Methodology, Investigation, Formal analysis, Data curation, Conceptualization. **Karl G. Wagner:** Writing – review & editing, Supervision, Resources, Project administration, Conceptualization. **Kristina E. Steffens:** Writing – review & editing, Supervision, Project administration, Methodology, Conceptualization.

## Declaration of generative AI and AI-assisted technologies in the writing process

During the preparation of this work, the authors used grammarly in order to check for grammar and spelling mistakes and improve the readability of the work. After using this tool, the authors carefully reviewed all edited content and take full responsibility for the content of this work.

## Funding statement

This research did not receive any specific grant from funding agencies in the public, commercial, or not-for-profit sectors.

## Declaration of competing interest

The authors declare the following financial interests/personal relationships which may be considered as potential competing interests:

Kristina E. Steffens reports equipment, drugs, or supplies was provided by Grace GmbH. Kristina E. Steffens reports equipment, drugs, or supplies was provided by JRS Pharma GmbH & Co. KG. Kristina E. Steffens reports equipment, drugs, or supplies was provided by Romaco Kilian GmbH. Kristina E. Steffens reports equipment, drugs, or supplies was provided by BASF SE. Kristina E. Steffens reports equipment, drugs, or supplies was provided by Peter Greven GmbH & Co KG. If there are other authors, they declare that they have no known competing financial interests or personal relationships that could have appeared to influence the work reported in this paper.

## Data Availability

Data will be made available on request.

## Appendix A Supplementary data

Supplementary material Image 1
