# Supplementary material for: Liquisolids as a platform for the formulation of cannabis tablets
Source: Int J Pharm X. 2026 Feb 24;11:100508. doi: 10.1016/j.ijpx.2026.100508 (PMC12955684; doi:10.1016/j.ijpx.2026.100508)
Supplement: Supplementary file 1 — Supplementary material [file mmc1.pdf]

Liquisolids as a Platform for the Formulation of Cannabis Tablets:

Supplementary material

Authors: Jan Appelhaus[1], Prof Dr. Karl G. Wagner[1], Dr. Kristina E. Steffens[1]*

[1]Department of Pharmaceutics, University of Bonn, Germany
Gerhard-Domagk-Str. 3, 53121 Bonn, Germany

*Corresponding author
E-mail: kristina.steffens@uni-bonn.de
Tel.: +49 228 735271, Fax: +49 228 735235

Other Authors
E-mail: karl.wagner@uni-bonn.de
Tel.: +49 228 735268

E-mail: j.appelhaus@uni-bonn.de
Tel.: +49 228 735509

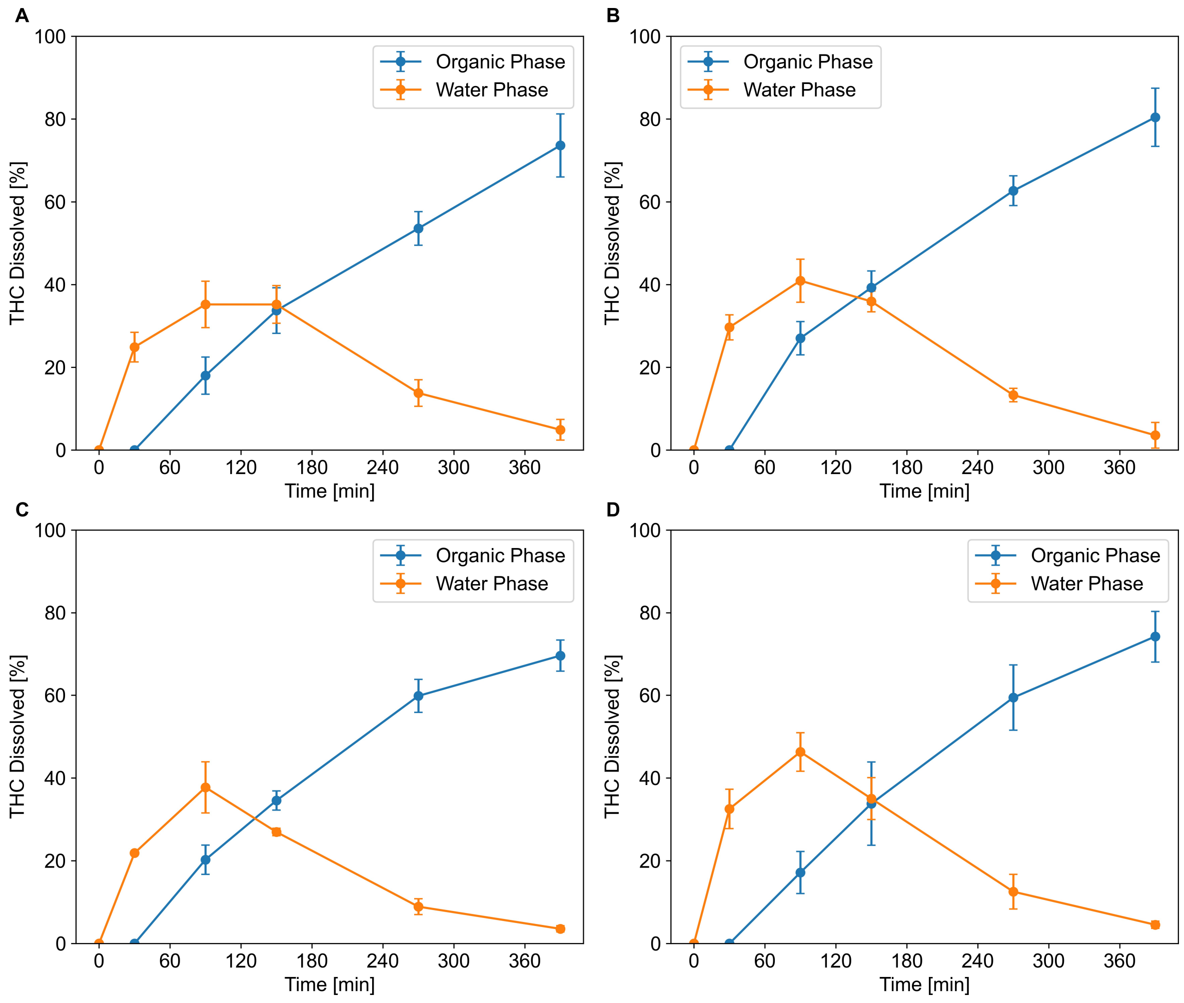

Figure S1 Biphasic dissolution behaviour of tetrahydrocannabinol (THC) after 0 (A), 7 (B), 30 (C) and 90 (D) days of air tight storage at 40 °C and 75 % humidity using FaSSIF at a pH of 5.0 as the aqueous and decanol as the organic phase

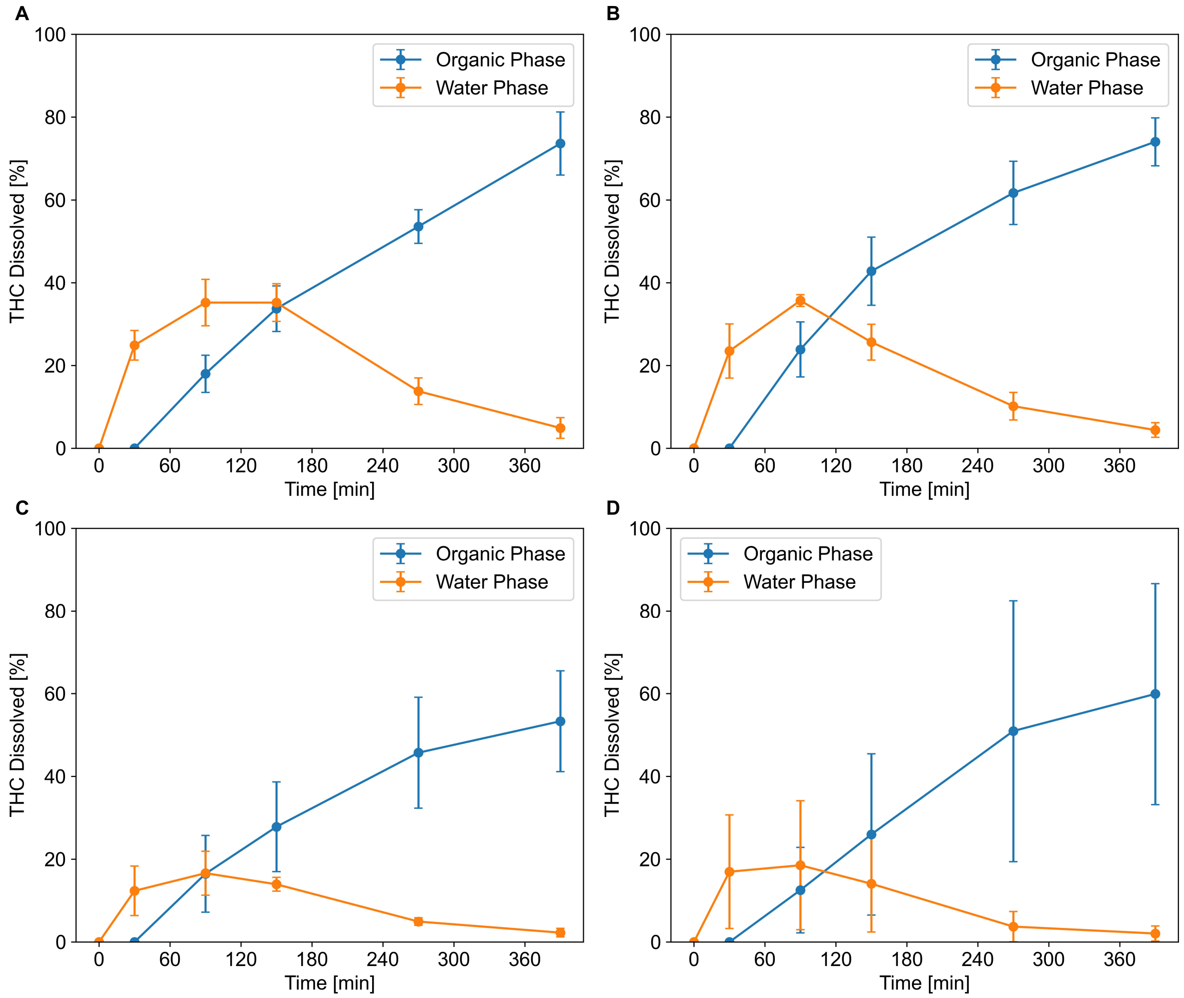

Figure S2 Biphasic dissolution behaviour of tetrahydrocannabinol (THC) after 0 (A), 7 (B), 30 (C) and 90 (D) days of open storage at 40 °C and 75 % humidity using FaSSIF at a pH of 5.0 as the aqueous and decanol as the organic phase

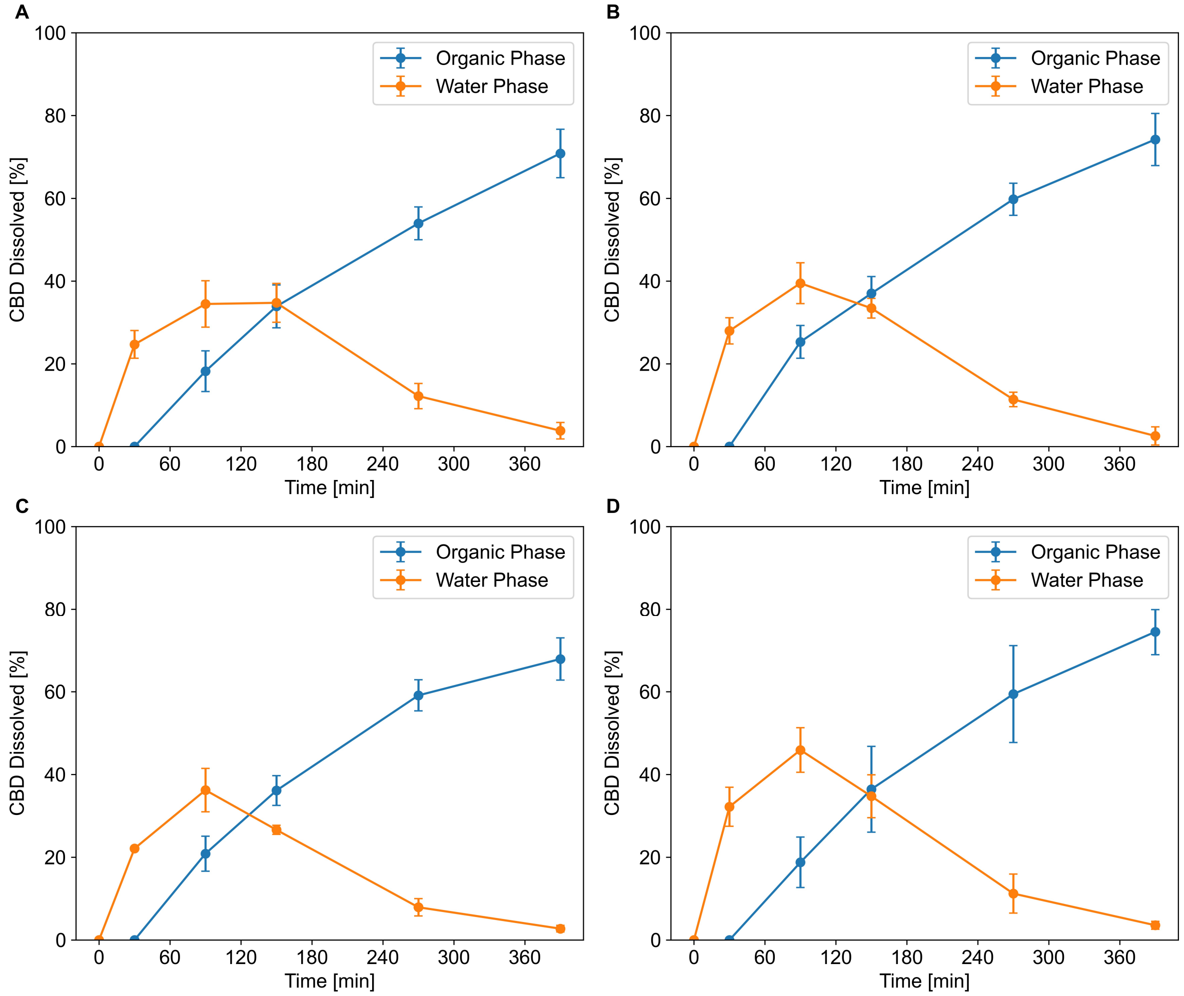

Figure S3 Biphasic dissolution behaviour of cannabidiol (CBD) after 0 (A), 7 (B), 30 (C) and 90 (D) days of air tight storage at 40 °C and 75 % humidity using FaSSIF at a pH of 5.0 as the aqueous and decanol as the organic phase

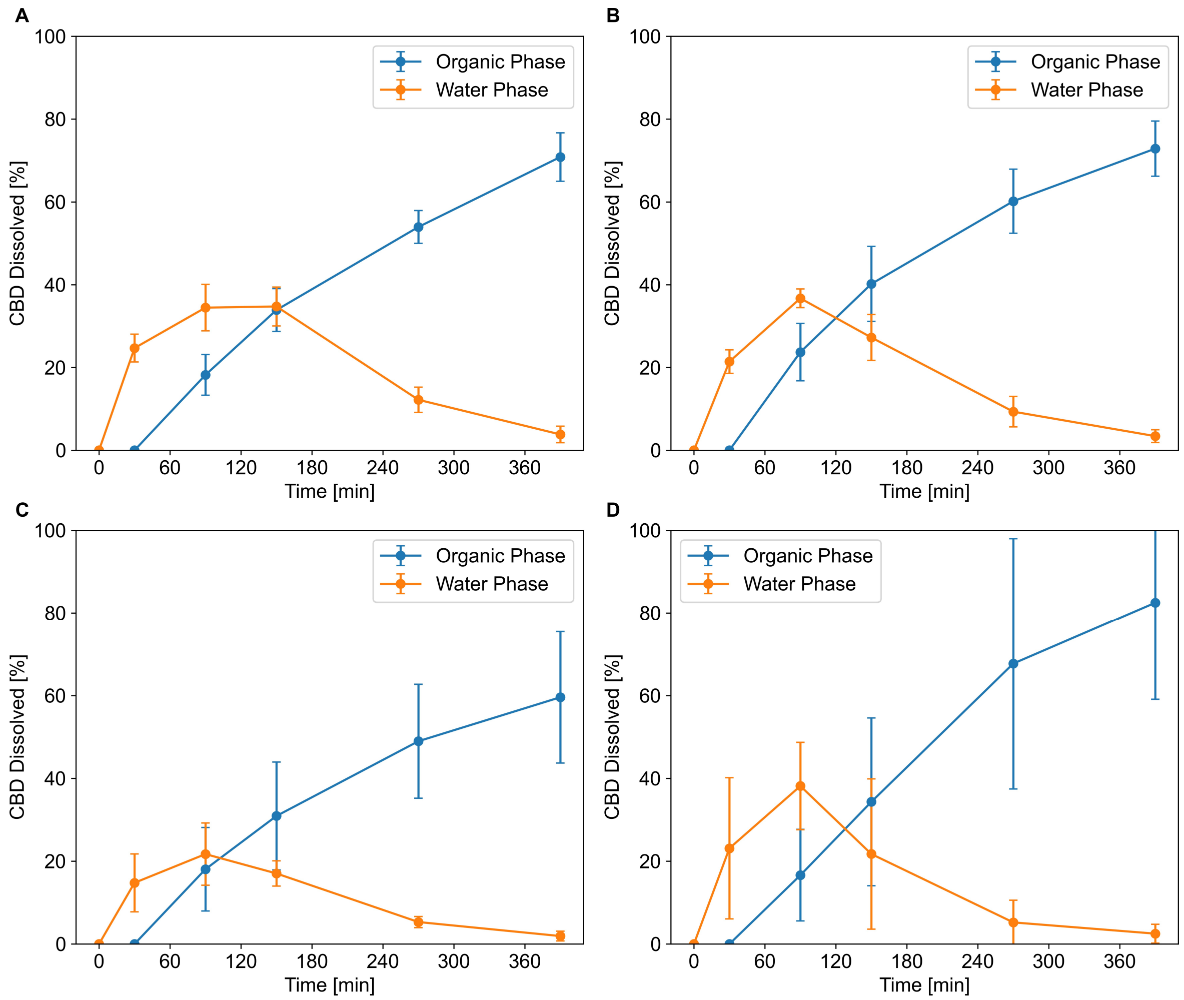

Figure S4 Biphasic dissolution behaviour of cannabidiol (CBD) after 0 (A), 7 (B), 30 (C) and 90 (D) days of open storage at 40 °C and 75 % humidity using FaSSIF at a pH of 5.0 as the aqueous and decanol as the organic phase
